# Role of Purinergic Receptor Expression and Function for Reduced Responsiveness to Adenosine Diphosphate in Washed Human Platelets

**DOI:** 10.1371/journal.pone.0147370

**Published:** 2016-01-25

**Authors:** Juergen Koessler, Stephanie Hermann, Katja Weber, Angela Koessler, Sabine Kuhn, Markus Boeck, Anna Kobsar

**Affiliations:** Institute of Transfusion Medicine and Haemotherapy, University of Wuerzburg, Wuerzburg, Germany; Medical Faculty, Ludwig Maximilians University Munich, GERMANY

## Abstract

**Background:**

Washing of platelets is an important procedure commonly used for experimental studies, e.g. in cardiovascular research. As a known phenomenon, responsiveness to adenosine diphosphate (ADP) is reduced in washed platelets, although underlying molecular mechanisms—potentially interfering with experimental results—have not been thoroughly studied.

**Objectives:**

Since ADP mediates its effects via three purinergic receptors P2Y1, P2X1 and P2Y12, their surface expression and function were investigated in washed platelets and, for comparison, in platelet-rich-plasma (PRP) at different time points for up to 2 hours after preparation.

**Results:**

In contrast to PRP, flow cytometric analysis of surface expression in washed platelets revealed an increase of all receptors during the first 60 minutes after preparation followed by a significant reduction, which points to an initial preactivation of platelets and consecutive degeneration. The activity of the P2X1 receptor (measured by selectively induced calcium flux) was substantially maintained in both PRP and washed platelets. P2Y12 function (determined by flow cytometry as platelet reactivity index) was partially reduced after platelet washing compared to PRP, but remained stable in course of ongoing storage. However, the function of the P2Y1 receptor (measured by selectively induced calcium flux) continuously declined after preparation of washed platelets.

**Conclusion:**

In conclusion, decreasing ADP responsiveness in washed platelets is particularly caused by impaired activity of the P2Y1 receptor associated with disturbed calcium regulation, which has to be considered in the design of experimental studies addressing ADP mediated platelet function.

## Introduction

Platelets play an important physiological role in hemostasis, wound healing or inflammation [1, 2]. They are also involved in pathophysiological processes, and in this regard, the analysis of platelet function is an important issue for the understanding of diseases like atherosclerosis, cancer or diabetes.

In experimental cardiovascular research, it is often required to provide fresh and functionally intact platelets, e.g. for the evaluation of molecular and celluar mechanisms leading to thrombogenesis or for pharmacological testing of antiplatelet drugs.

However, the isolation and preparation of platelets, especially of washed platelets, are laborious and time-consuming procedures. Besides, the quality of platelets has to be warranted in order to rule out impeding influences on experimental results caused by inadequate handling. Washing steps and short-time storage should not affect the functional integrity of platelets.

The inhibition of adenosine diphosphate (ADP) induced aggregation is an important pharmacological principle for the treatment of cardiovascular diseases, e.g. after stent implantation in coronary heart disease. The investigation of ADP mediated platelet function and signaling has to be based on reliable experimental procedures. However, as a known phenomenon, responsiveness to ADP is rapidly tampered in washed platelets [3, 4]. The mechanisms contributing to that phenomenon are not well understood and have not yet been thoroughly studied.

ADP is a physiological platelet activator mediating its effects via purinergic receptors. Platelets contain three different purinergic receptors: P2Y1, P2Y12 and P2X1. Two of them, P2Y1 and P2Y12, are guanine nucleotide-binding-protein (G-protein) coupled receptors [5, 6, 7], whereas P2X1 is an adenosine triphosphate (ATP)-gated, non-selective cation channel. P2Y1 is a G_q_-coupled receptor, activating platelet phospholipase C and stimulating calcium release from intracellular stores [8, 9]. P2Y12 inhibits platelet adenylyl cylase through G_αi_ [8, 9]. Simultaneous activation of both P2Y1 and P2Y12 results in platelet aggregation. Stimulation of the P2X1 receptor alone causes a rapid calcium influx in platelets that can synergize P2Y1 effects [10] and induce platelet shape change, but is not able to induce platelet aggregation [11].

The aim of this study was to investigate the effects caused by the critical process of washing platelets using CGS (sodium **c**hloride, D-**g**lucose, tri**s**odium citrate) buffer [12, 13, 14] on ADP dependent platelet function. ADP induced aggregation was measured after washing and short-time storage of platelets for 2 hours in HEPES containing buffer in comparison to platelet-rich plasma (PRP). In addition, the effects on expression and function of platelet purinergic receptors were studied in order to explore underlying mechanisms of affected ADP mediated platelet aggregation.

## Materials and Methods

### Materials

ADP was from Haemochrom Diagnostica GmbH (Essen, Germany). FITC-conjugated goat anti-rabbit polyclonal antibody, prostaglandin E1 (PGE1), Acetylsalicylic acid (ASS), Probenecid, Pluronic^®^ F-127, 4-[2-**h**ydroxy**e**thyl]-1-**p**iperazine**e**thane**s**ulfonic acid (HEPES) and apyrase were from Sigma-Aldrich Chemie GmbH (Muenchen, Germany). Rabbit polyclonal anti-P2Y1, anti-P2Y12 and anti-P2X1 antibodies were from Alomone Labs (Jerusalem, Israel). The selective P2Y1 receptor agonist [(1*R*,2*R*,3*S*,4*R*,5*S*)-4-[6-Amino-2-(methylthio)-9*H*-purin-9-yl]-2,3-dihydroxy- bicyclo[3.1.0] hex-1-yl]methyl] diphosphoric acid mono ester trisodium salt (MRS2365); the selective antagonist of P2Y1 (1R*,2S*)-4-[2-Iodo-6-(methylamino)-9*H*-purin-9-yl]-2-(phosphor nooxy)bicyclo[3.1.0]hexane-1-methanol dihydrogen phosphate ester tetraammonium salt (MRS2500), the agonist of P2X1 receptor α,β-Methyleneadenosine 5'-triphosphate trisodium salt (α,β-MeATP), and the potent P2X1 antagonist 4,4',4'',4‴-[Carbonylbis(imino-5,1,3-benzenetriyl-*bis*(carbonylimino))] *tetrakis*-1,3-benzenedisul—fonic acid, octasodium salt (NF449) were from R&D Systems GmbH (Wiesbaden-Nordenstadt Germany). Fluo-4A M Cell permeant was from Life Technologies GmbH (Darmstadt, Germany).

### Blood collection

Venous whole blood was obtained from informed healthy voluntary donors. Blood was collected in polystyrene tubes containing 3.2% citrate buffer (106 mM trisodium citrate, Sarstedt, Nuembrecht, Germany). Our studies with human platelets and the consent procedure were approved by our local ethics committee of the University of Wuerzburg (approval number 47/12). The participants provided their written informed consent to participate in this study. The study was performed according to our institutional guidelines and to the Declaration of Helsinki.

### Preparation and stimulation of PRP and washed human platelets

Washed platelets were prepared as described [15]. Briefly, 3 mM EGTA was added to whole blood to prevent platelet activation. PRP was obtained by centrifugation at 280 g for 5 minutes (min).

For the preparation of washed platelets, PRP was centrifuged at 430 g for 10 min. The pelleted platelets were washed once in CGS buffer (120 mM sodium chloride, 12.9 mM trisodium citrate, 30 mM D-glucose, pH 6.5) [12, 13, 14] and resuspended in HEPES buffer (150 mM NaCl, 5 mM KCl, 1 mM MgCl2, 10 mM D-glucose, 10 mM HEPES, pH 7.4) [16] to a final concentration of 3 x 10^8^ platelets/mL.

Directly after preparation, the platelet suspension or PRP were aliquoted in Eppendorf tubes (30 μL per tube) and stored at room temperature (RT) without shaking. After resting for 15 min (representing the first time point—0 min), platelets were used for experiments. Other aliquots were stored for 30, 60 and 120 min to investigate time dependent effects.

### Flow cytometric analysis of human platelets

30 μL of washed human platelets (3 x 10^8^ platelets/mL) or PRP were preincubated with 2.7 μl of anti-purinergic receptor antibodies (0.8–1.0 mg/mL) for 15 min at RT followed by 15 min incubation at 37°C. Samples were stopped with 0.1% formaldehyde, fixed for 10 min at RT and centrifuged for 2 min at 20000 g. The pellet was resuspended in 100 μl of PBS/5 mM glucose/0.5% BSA and stained for 25 min with 1 μl of FITC-conjugated goat anti-rabbit antibody. Finally, the samples were diluted with 500 μL of PBS/5 mM glucose/0.5% BSA, and analyzed on a FACS Calibur flow cytometer from Becton Dickinson (Franklin Lakes, NJ, USA) using CELLQuest software, version 6.0. The platelet population was identified by its forward and side scatter distribution and 10000 events were analyzed for mean fluorescence. Data from five independent experiments were used for statistical analysis.

### Platelet aggregation

Platelet aggregation was measured using an APACT 4004 aggregometer (LabiTec, Ahrensburg, Germany). Washed human platelets (3 x 10^8^ platelets/mL) supplemented with 1 mM CaCl_2_ or PRP were stimulated with HEPES buffer or 10 μM ADP. Platelet aggregation was measured for 5 min under continuous stirring at 1000 rpm and 37°C. Data from five independent experiments were used for statistical analysis.

### Platelet preparation for the measurement of P2Y1 activity

To prepare platelets for P2Y1 activity measurement, 500 nM PGE1 was added to the PRP. PRP was centrifuged at 430 g for 10 min and the pellet was washed with 5 mL of modified Tyrode buffer (10 mM HEPES, 150 mM NaCl, 3 mM KCl, 1 mM MgCl_2_, 5 mM glucose, and 0.1% BSA, pH 6.5) containing 500 nM PGE1. Platelets were resuspended in modified Tyrode buffer without PGE1 and platelet concentration was adjusted to 0.6 x10^8^ platelets/mL [17].

### Platelet preparation for the measurement of P2X1 activity

To prepare platelets for P2X1 activity measurement, 1 mM aspirin and 0.3 U/mL apyrase were added to the PRP. PRP was centrifuged at 430 g for 10 min and the pellet was washed with 5 mL of modified Tyrode buffer containing 1 mM aspirin and 0.3 U/mL apyrase. Platelets were resuspended in modified Tyrode buffer containing 0.3 U/mL apyrase and platelet concentration was adjusted to 0.6 x10^8^ platelets/mL [17].

### Measurement of P2Y1 and P2X1 activity

The activity of platelet purinergic P2Y1 and P2X1 receptors was measured by fluorescence induced by calcium flux in Fluo-4AM loaded platelets after selective stimulation [17]. Briefly, in each well of a 96-well black plate, 100 μL of washed platelets were mixed with an equal volume of Hank’s buffered saline solution (HBSS) containing 10 mM HEPES, 0.1% BSA, 2.5 mM probenecid, 1 mM EGTA, 0.01% pluronic acid and 2 μM Fluo-4AM at pH 7.4. For P2X1 measurements, EGTA was substituted by 2.5 mM calcium and apyrase was added to the final concentration of 0.3 U/mL. The plate was incubated for 20 min at RT in the dark, followed by 20 min incubation at 37°C. During the last 10 min of incubation, 2 μL of 100 μM MRS2500, a P2Y1 antagonist, or 2 μL of 100 μM NF 449, a P2X1 antagonist, were added. After measurement of the basal fluorescence (Ex 488—Em 538; 20 measurements at 1 s), platelets were stimulated with 2 μL of 100 μM MRS2365, a P2Y1 agonist, or 2 μL of 100 μM α,β-MeATP, a P2X1 agonist. After stimulation, fluorescence values were measured every s for the next 3 min. Fluorescence signals were measured and analyzed by Fluoroscan Ascent Microplate Fluorometer from Fisher Scientific GmbH (Schwerte, Germany). Data from five independent experiments were used for statistical analysis.

### Measurement of P2Y12 activity

The activity of platelet P2Y12 receptor was measured by the flow cytometric PLT VASP/P2Y12 Kit (Stago, Asnières sur Seine, France). For the investigation of time dependent changes of P2Y12 receptor activity, aliquots of WB and washed platelets stored for 0, 30, 60 and 120 min at RT were stimulated with PGE1 alone or with a combination of PGE1 and ADP. After stimulation, samples were fixed and stained as described in the manufacturer's instructions, followed by flow cytometric measurement of fluorescence induced by phosphorylated vasodilator-stimulated phosphoprotein (VASP). Platelet reactivity index (PRI) was calculated using corrected mean fluorescence intensities (MFIc) as PRI = [MFIc (PGE1)–MFIc (PGE1 + ADP)] / [MFIc (PGE1)] x 100%. Data from eight independent experiments were used for statistical analysis.

### Statistical analysis

Data are presented as mean ± standard error of the mean (SEM). The n-values refer to the number of experiments, each made with different blood donors. Differences between groups were analyzed by paired and un-paired Student's t-test as appropriate using MedCalc statistic program (MedCalc Software bvba, Mariakerke, Belgium). P<0.05 was considered statistically significant.

## Results

### ADP induced platelet aggregation continuously decreases in washed platelets after preparation

**Fig 1:**
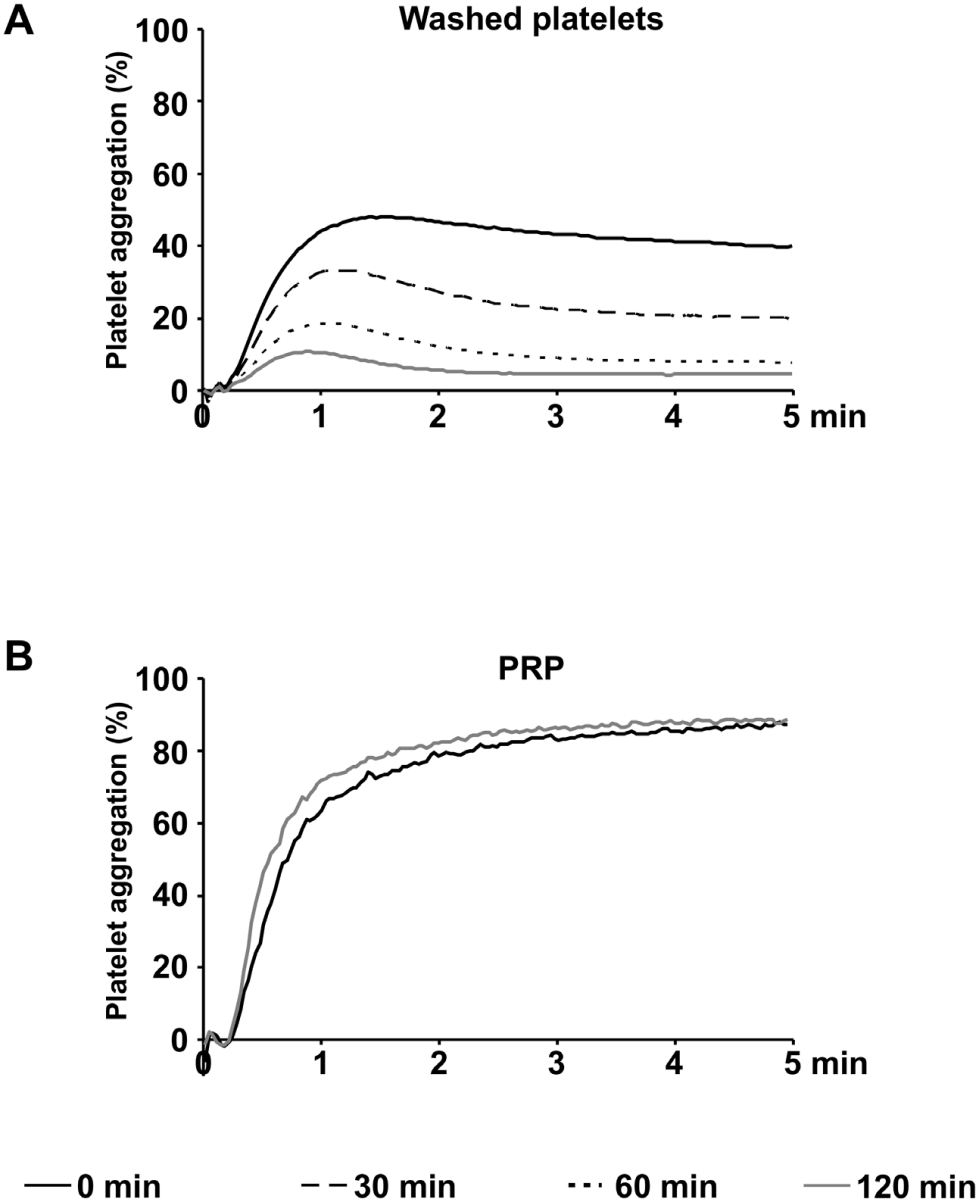
ADP induced aggregation continously decreases in washed platelets after preparation. Light transmission aggregometry induced with 10 μM ADP was measured in washed platelets (3 x 10^8^ per mL resting in HEPES buffer at RT) (A) and in PRP (B) at different time points after preparation as indicated. Mean aggregation traces of five independent experiments are presented.

In freshly prepared washed platelets, suspended and resting in HEPES buffer at RT, maximal platelet aggregation induced by 10 μM ADP reached values of 50.2%±4.0%. In the course of time, aggregation continuously decreased to 33.3%±2.3% after 30 min, to 18.7%±3.9% after 60 min and to 10.8%±2.8% after 120 min (Fig 1A). In contrast to washed platelets, in PRP resting at RT without agitation, maximal ADP induced aggregation showed values of 87.1%±4.7% directly after preparation and was completely maintained after 120 min with 88.5%±4.3% (Fig 1B).

### Washed platelets, but not platelets in PRP, show a biphasic variation of purinergic receptor expression after preparation

**Fig 2:**
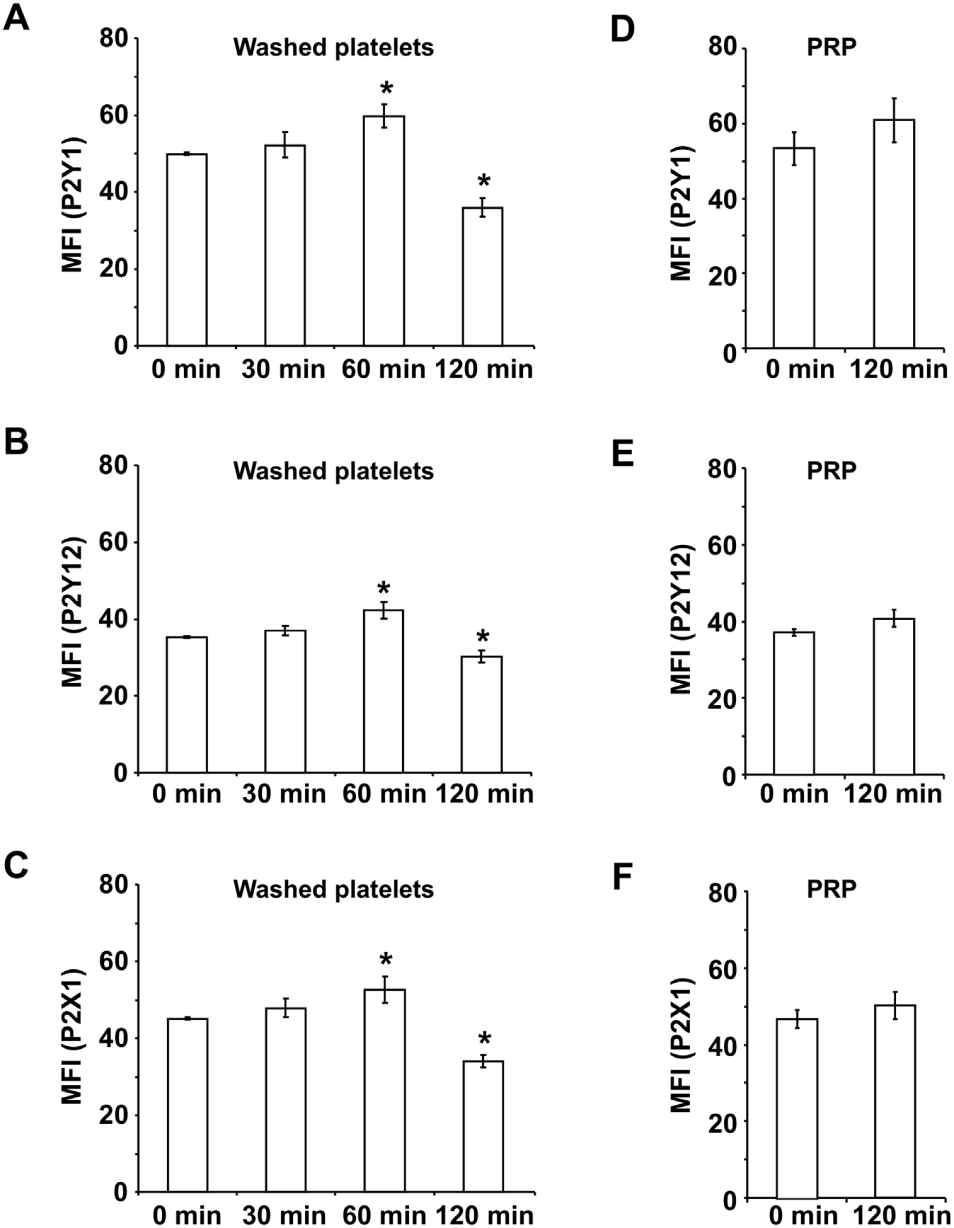
The surface expression of platelet purinergic receptors show a biphasic variation in washed platelets after preparation. Washed platelets (3 x 10^8^ per mL resting in HEPES buffer at RT) and platelets in PRP were stained with purinergic receptor specific antibodies at different time points after preparation as indicated. The basal receptor surface expression was quantified by flow cytometry. The histograms show the mean fluorescence intensities (MFI) of P2Y1 (A and D), P2Y12 (B and E) and P2X1 (C and F). Results are presented in absolute arbitrary units as mean ± SEM; n = 5; *: p < 0.05 (compared to 0 min).

Flow cytometric analysis of washed platelets revealed that the basal surface expression of all investigated platelet purinergic receptors P2Y1, P2Y12 and P2X1 changed in a biphasic manner. First, the amount of receptors grew continuously reaching maximal values 60 min after preparation, followed by a significant reduction after 120 min (Fig 2A–2C).

At 60 min, mean fluorescence of P2Y1 expression was increased by 19.7%±6.3%, whereas at 120 min, it was diminished by 27.9%±4.7% compared to freshly prepared washed platelets (Fig 2A). The surface expression of P2Y12 was elevated by 19.9%± 6.3% and of P2X1 by 16.6%±7.4% at 60 min (Fig 2B and 2C). In contrast, the receptor density was reduced by 14.3%±4.4% for P2Y12 and by 24.7%±3.8% for P2X1 after 120 min compared to 0 min.

In PRP, there were no significant differences in the surface expression of purinergic receptors during the observed time period of 120 min, although the values showed a slightly increasing tendency (Fig 2D–2F).

### The functional activity of the P2Y1 receptor decreases continuously in washed platelets, but not in platelets from PRP

**Fig 3:**
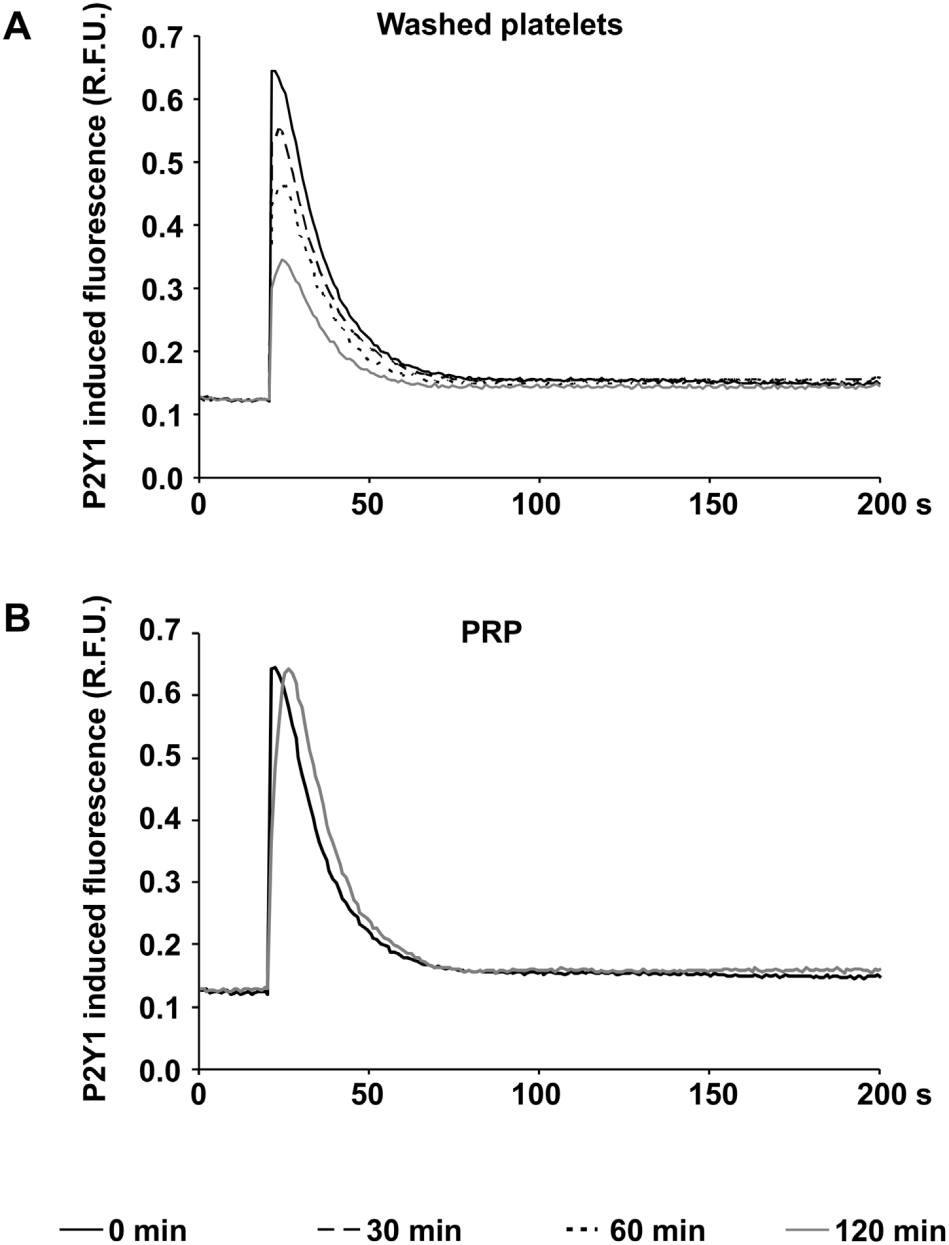
P2Y1 function continuously decreases in washed platelets. The figures show the calcium induced fluorescence curves in Fluo-4AM loaded washed platelets (A) and in platelets from PRP (B) after stimulation with the P2Y1 agonist MRS2365 at different time points after preparation as indicated. Mean fluorescence curves of five independent experiments are presented (relative fluorescence units, R.F.U.).

The functional activity of P2Y1 receptors was measured by calcium induced fluorescence in Fluo-4AM loaded platelets stimulated with MRS2365, a selective agonist of the P2Y1 receptor. In freshly prepared washed platelets, 1 μM MRS2365 induced a rapid and more than 5.2 fold increase of calcium induced fluorescence compared to basal levels (Fig 3A). In the course of time after preparation, the induced calcium flux continuously decreased to 4.3 fold, 3.5 fold or 2.7 fold elevations after 30, 60 and 120 min.

In platelets from fresh PRP, a 5.2 fold increase of calcium induced fluorescence was observed and comparable with the values obtained 120 min after preparation of PRP (Fig 3B).

### Affected response to PGE1 causes decreasing PRI values in washed platelets after preparation

**Fig 4:**
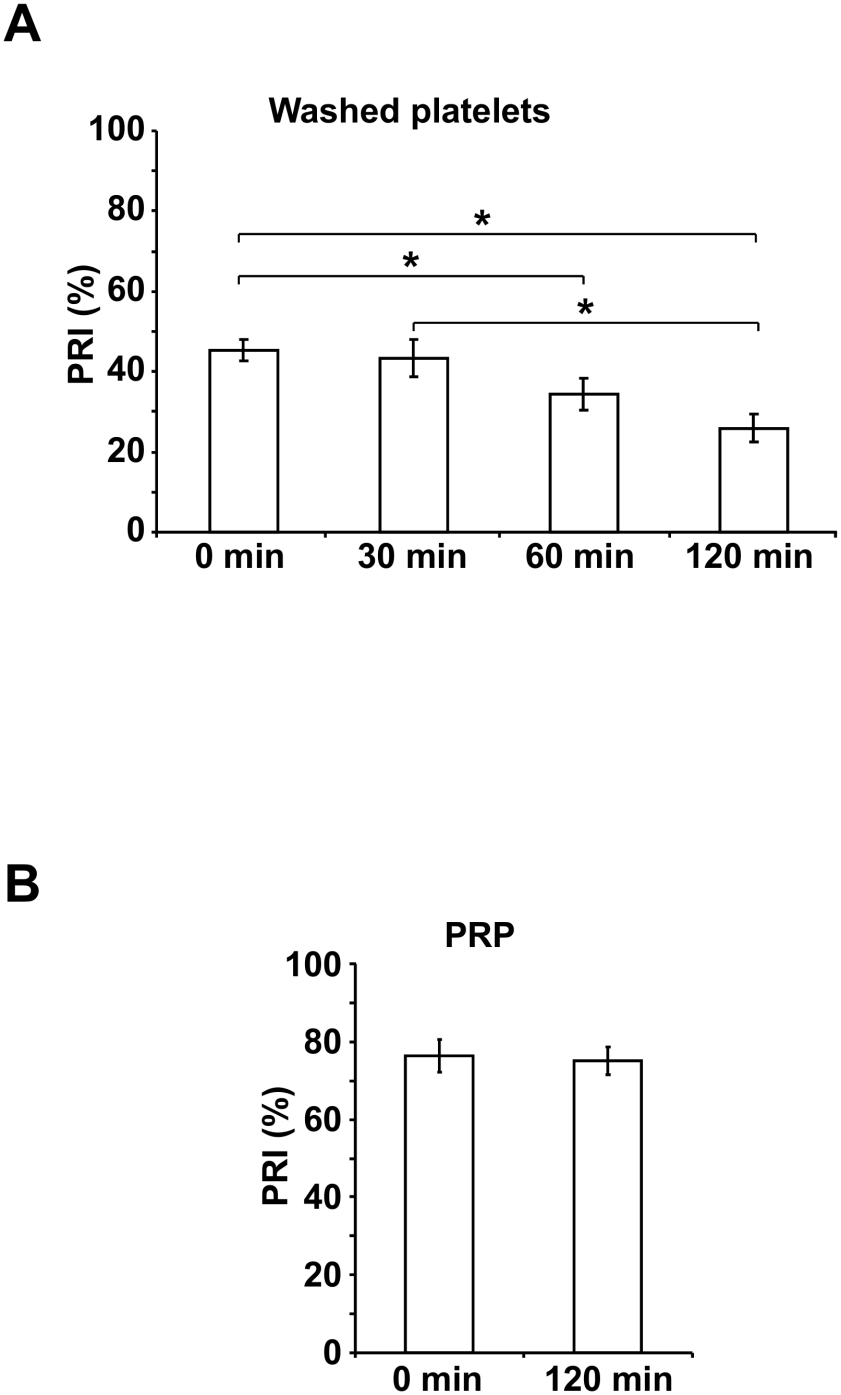
PGE1-stimulated VASP phosphorylation decreases in washed platelets resulting in reduced PRI values. The platelet reactivity index (PRI) was determined by flow cytometric measurement of VASP phosphorylation stimulated with PGE1 alone or with ADP and PGE1 in washed platelets or in platelets from PRP at different time points after preparation as indicated. The histograms show the PRI values in washed platelets (A) and in PRP (B) presented as mean ± SEM (%); n = 8; *: p < 0.05 (compared to 0 min).

Freshly prepared washed platelets showed a mean PRI value of 45.3%±2.8% (Fig 4A). For 30 min after preparation, the PRI value remained stable with 43.3%±4.8%, while the PRI dropped significantly to 34.4%±4.1% at 60 min and to 25.9%±3.5% at 120 min.

In contrast, the PRI values of platelets remained unaffected in freshly prepared PRP with values of 76.4%±4.2 and 120 min after preparation with values of 75.1%±3.6% (Fig 4B).

The analysis of the originally measured MFIc values revealed that the MFIc of PGE1 stimulated washed platelets decreased. The mean MFIc values stimulated with the combination of PGE1 and ADP, however, were almost unchanged for 120 min after preparation of washed platelets (Table 1).

**Table 1:** MFIc values of VASP phosphorylation in washed platelets.

Storage Time	MFIc	
	PGE1 alone (mean±SEM)	PGE1 with ADP (mean±SEM)
0 min	52.2±4.9	30.0±2.7
30 min	46.9±5.5	27.0±2.6
60 min	38.0±4.7*	25.1±3.0
120 min	37.5±2.9*	29.5±2.9

* P < 0.05, compared to freshly prepared washed platelets; n = 8.

### The functional activity of P2X1 remains stable in washed platelets and PRP

**Fig 5:**
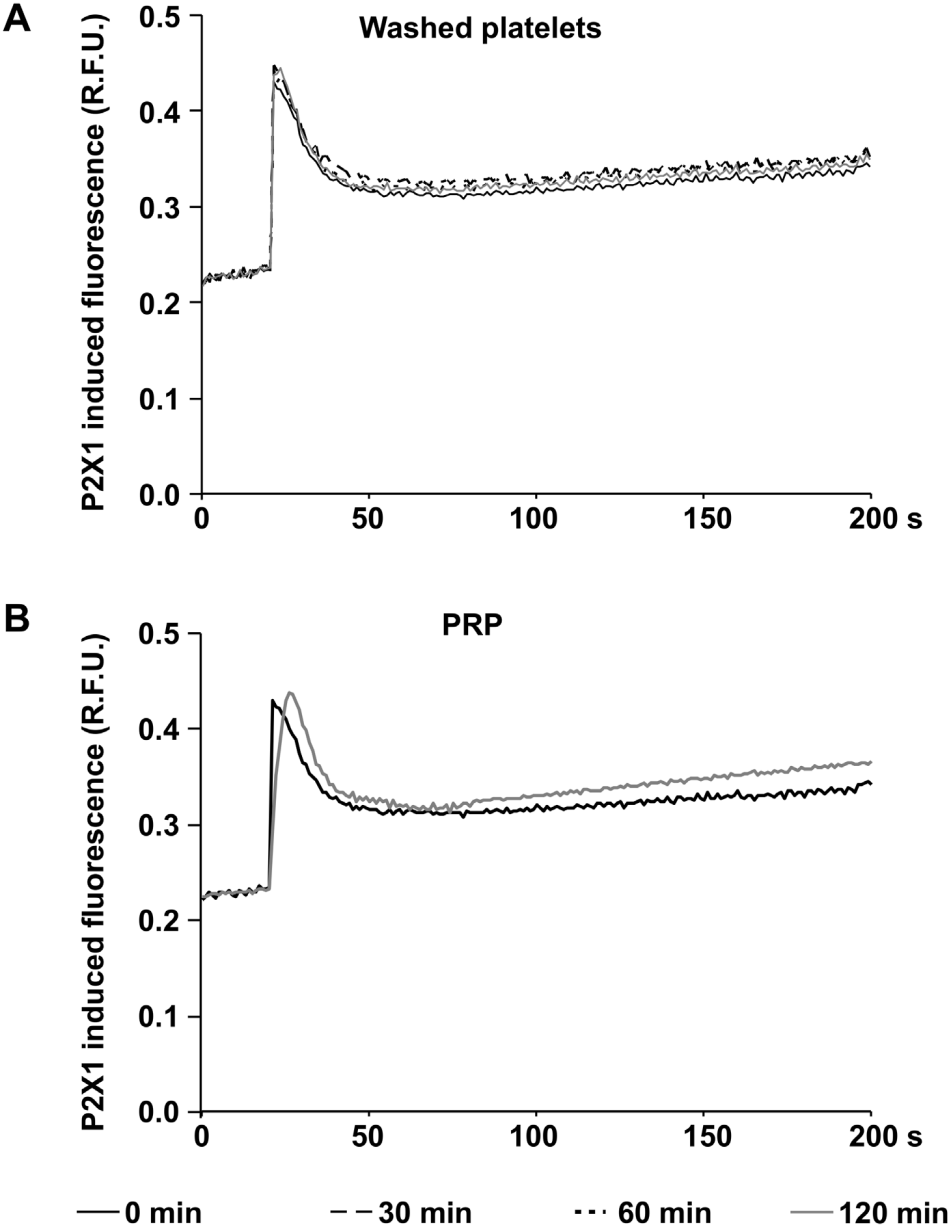
P2X1 function remains stable in washed platelets and in PRP. The figures show the calcium induced fluorescence curves in Fluo-4AM loaded washed platelets (A) and in platelets from PRP (B) after stimulation with the P2X1 agonist α,β-MeATP at different time points after preparation as indicated. Mean fluorescence curves of five independent experiments are presented (relative fluorescence units, R.F.U.).

The functional activity of P2X1 receptors was assessed by calcium induced fluorescence in Fluo-4AM loaded platelets stimulated with α,β-MeATP, a selective agonist of the P2X1 receptor. The stimulation with 1 μM α,β-MeATP induced a rapid and almost two fold increase of calcium induced fluorescence in freshly prepared washed platelets (Fig 5A) and in platelets from PRP (Fig 5B). These values remained stable for 120 min after preparation of washed platelets (Fig 5A) and PRP (Fig 5B).

## Discussion

Washing procedures are critical steps in the preparation of platelets for experimental research, since integrity and function of platelets may be affected. This study investigated the initial effects of washing platelets on ADP-mediated platelet responsiveness in comparison with freshly prepared PRP in order to evaluate molecular and functional alterations in washed platelets. As a common medium, CGS buffer was used for the preparation of washed platelets [12, 13, 14]. Subsequent storage of PRP and of washed platelets (suspended in HEPES buffer) was performed under equal conditions at 22°C without agitation.

10 μM ADP, a dose frequently used for clinical investigations in PRP [18], was still able to induce aggregation in washed platelets. However, maximal values were reduced by 50% directly after preparation and continuously decreased during the course of 2 hours in contrast to PRP. Since the effects of ADP are mediated via purinergic receptors [5, 6, 7], it was of interest to measure membrane expression and function of platelet purinergic receptors in washed platelets and, for comparison, in PRP.

Surprisingly, the surface expression of P2Y1, P2Y12 and P2X1 receptors continuously rose in washed platelets during the first 60 min after preparation. Such an increase of surface expression of different platelet receptors is commonly observed in preactivated platelets [19, 20, 21]. Extended storage, however, resulted in a significant reduction of purinergic receptor expression, indicative of enhanced degenerative processes in plasma-free medium, since surface expression did not change in PRP even after 120 min of storage.

The transient increase of surface expression of purinergic receptors after washing of platelets is in contradiction to the continuous decrease of ADP induced aggregation, which indicates that tampered functional activity of purinergic receptors—and not the absolute amount—may be responsible for the observed phenomenon.

The regulation of the cytosolic calcium concentration is crucial for the activation of signaling pathways necessary for platelet aggregation [6, 9, 11]. It is known that the activation of the P2Y1 and the P2X1 receptor leads to an elevation of intracelluar calcium levels in platelets [6, 9, 10, 11]. During storage, the increment of the cytosolic calcium concentration induced by the P2Y1 agonist MRS2365 permanently decreased in washed platelets, but not in PRP, pointing to a functional loss of this receptor in the absence of plasma. In contrast, the P2X1 receptor appears to play a minor role in this context, since calcium influx was maintained in both stored washed platelets and in PRP after selective stimulation of the receptor.

The flow cytometric platelet VASP/P2Y12 assay based on ADP induced inhibition of PGE1 induced cyclic adenosine monophosphate (cAMP)-mediated VASP phosphorylation and presented as PRI is a common method to measure the function of the P2Y12 receptor [15, 22]. The PRI values in PRP, freshly prepared and stored for 120 min were within reference levels of healthy individuals [23], suggesting that the functional activity of P2Y12 in PRP is not affected by preparation and storage of PRP for 120 min. In contrast, the basal PRI values in freshly prepared washed platelets were generally lower than in PRP pointing to a partial loss of activity due to platelet washing. Since the PRI is a calculated quantity, the corresponding MFIc values after PGE1 stimulation alone and combined ADP/PGE1 stimulation were additionally analyzed. MFIc values of both PGE1- and PGE1/ADP- stimulated platelets were stable for 30 min after preparation resulting in similar PRI values compared to freshly washed platelets. During ongoing storage, the PRI values continuously decreased, which is particularly caused by reduced PGE1-stimulated VASP phosphorylation in washed platelets. ADP induced inhibition of VASP phosphorylation via the P2Y12 receptor, however, was not affected suggesting that the P2Y12 function itself was maintained during the two hours after preparation, whereas PGE1 responsiveness began to decline.

In conclusion, this study revealed for the first time underlying molecular and functional mechanisms influencing ADP mediated platelet function after preparation of washed platelets. These novel data show that calcium dependent signaling, and consecutively, ADP induced aggregation is affected in washed platelets, but not in platelets stored as PRP for 2 hours confirming earlier studies with stable aggregation in PRP up to 4 hours [24]. Especially the continuous decline of the P2Y1 receptor function is an important mechanism resulting in impaired calcium regulation of washed platelets. During continued storage for more than 30 min, descending receptor surface expression additionally contributes to tampered ADP mediated platelet responsiveness after the washing procedure.

As a limitation of the study it must be considered that these results refer to procedures using CGS buffer for washing platelets and using HEPES buffer as storage medium, although these conditions are frequently used for experimental platelet research. Under these conditions, it is advisable to perform studies addressing ADP mediated platelet function preferably with freshly washed platelets or at least within 30 min after washing to rule out severe preanalytical errors.

For some purposes, it would be of advantage to have an opportunity to store washed platelets for a longer period of time, e.g. in laborious experiments or in transfusion medicine to provide platelet concentrates without plasma for patients suffering from allergic reactions [25, 26].

However, the presence of plasma is obviously required to preserve ADP responsiveness of platelets *in vitro*, which was also demonstrated by studies using synthetic storage media [27], whereas it remains unclear which of the plasma components are essential or possibly substitutable. Previous reports showed that ADP-degrading activities of plasma may play a role for the prevention of purinergic receptor desensitisation [28] and for the preservation of purinergic receptor functionality [28, 29]. Therefore, further studies are needed addressing different washing and storage conditions for the optimization of washed platelet preparation to maintain platelet quality.
